# Experimental and Analytical Modeling of Flexural Impact Strength of Preplaced Aggregate Fibrous Concrete Beams

**DOI:** 10.3390/ma15113857

**Published:** 2022-05-28

**Authors:** Gunasekaran Murali, Sallal Rashid Abid, Nikolai Ivanovich Vatin

**Affiliations:** 1 Peter the Great St. Petersburg Polytechnic University, 195251 Saint Petersburg, Russia; vatin@mail.ru; 2 Civil Engineering Department, Wasit University, Kut 52003, Iraq; sallal@uowasit.edu.iq

**Keywords:** PAFC beam, fibers, impact energy, failure, grout, beam size, modeling

## Abstract

Preplaced aggregate fibrous concrete (PAFC) is a revolutionary kind of concrete composite that is gaining popularity and attracting the interest of academics from across the world. PAFC is a uniquely designed concrete prepared by stacking and packing premixed fibers and coarse aggregate in a steel mold. The gaps between the fibers and aggregates are subsequently filled by injecting a cement grout with high flowability. This study investigates the impact performance of three different sizes of PAFC beams. Steel and polypropylene fibers were used in a 3% dosage to make three different beam sizes, measuring 550 × 150 × 150 mm, 400 × 100 × 100 mm, and 250 × 50 × 50 mm. According to ACI Committee 544, all beams were subjected to a drop weight flexural impact test. Compressive strength, impact energies at initial crack and failure, ductility index, and failure mode were evaluated. Additionally, analytical modeling was used to compute the failure impact energy for the fibrous beams. The results showed that the addition of fibers increased the capacity of the tested beams to absorb greater flexural impact energy. Compared to polypropylene fibers, steel fibers had better crack propagation and opening resistance because of their higher tensile strength and crimped and hooked end configuration. For all large-size beams, the analysis of the percentage increase in impact energy at the failure stages was found to be 5.3 to 14.6 times higher than the impact energy at cracking.

## 1. Introduction

A civil engineering structure will be exposed to a broad range of loads over its lifespan. Impact loads are severe loading instances that are very unlikely to occur throughout the lifespan of a structure [1]. Although terrorist attacks have escalated in recent decades, impact analysis has become critical to guarantee that buildings are secure. Impact loading scenarios include the influence of falling items on industrial flooring [2], impacts from a ship or ice collisions on the seafloor and offshore structures [3], accidents involving vehicles and bridges or structures [4] and nuclear containment facilities that are affected by airplanes and missiles [5]. As a result of the confined and transitory nature of impact loading, structural elements exposed to it may react differently from those under a static load. Additionally, the dynamic characteristics of materials may vary from those under static loading. Therefore, it is essential to examine the different building materials’ performance due to technological development and material innovations.

Preplaced aggregate concrete (PAC) is also called grouted aggregate concrete and two-stage concrete. An unorthodox technique is used to manufacture a unique form of concrete that differs from conventional concrete. When making conventional concrete, the components are thoroughly mixed before being poured into the molds. Preplaced coarse aggregate is piled into the mold, followed by grout injection, filling in any spaces between the coarse aggregate particles and forming a monolithic structure [6]. This may reduce a cement paste volume by 50%, resulting in lower cement usage and thus lower greenhouse gas emissions. A monolithic and dense structure may be achieved without the use of consolidation processes such as compaction or vibration [7]. Grouting for PAC applications may be carried out in one of two ways: either by gravity or by a pump. In the gravity grouting method, the grout is poured on top of the piled aggregate and allowed to penetrate through the aggregate body under gravity. Grouting narrow sections with a depth under 300 mm is especially well-suited to this technique [8]. A network of pipes pump the grout into the aggregate mass from the bottom up during the pumping procedure. PAC is beneficial in various instances, including nuclear power plants, radiation protective buildings, lower shrinkage tunnels, dams made of mass concrete with a low heat of hydration, and underwater construction [9]. It may also be used to restore concrete and masonry structures, which is an additional benefit. The high rigidity and low tensile strength of PAC components are the primary difficulties that have led to the classification of concrete as a brittle material such as conventional concrete. PAC must have great strength and outstanding durability in the applications mentioned earlier. Because of this, new building materials that may improve the concrete ductility are in high demand. As a result, adding fibers to the concrete mixture may be viable for achieving the desired qualities. Tahenni et al. [10] reported that the steel fibers do not significantly alter ordinary concrete’s compressive strength or modulus of elasticity. This improvement is especially noticeable when the fiber content (2% for plain concrete) is increased. Tahenni et al. [11] reported that the transverse reinforcement effectively reduces diagonal crack widths and reduces post-cracking loads. The onset of diagonal cracks is postponed, and their breadth is greatly reduced. Li et al. [12] reported that the compressive strength is enhanced by the more uniform distribution of the 13 mm short straight steel fibers in the ultra-high-performance fibrous matrix. The 30 mm hook-ended or 60 mm long 5D fibers, on the other hand, have a greater influence on reinforcing tensile and flexural strength and impact resistance.

Much research was conducted to evaluate the mechanical properties of PAFC. According to the research of Rajabi et al. [6], preplaced aggregate concrete outperformed ordinary concrete in terms of compressive strength, tensile strength, Young’s modulus, ultrasonic pulse velocity, and Schmidt hammer rebound number. Lv et al. [13] reported that the preplaced aggregate concrete’s cubic compressive strength, splitting tensile strength, and elastic modulus all decreased when the ratio of water to binder and sand to binder increased. The elastic modulus of preplaced aggregate concrete might be increased by up to 20% when compared to conventional concrete at identical compressive strengths. Alrshoudi et al. [14] reported that the compressive strength of PAFC specimens was reduced when waste polypropylene fibers were added to the mixture for both the gravity and pumping method of grout injection. The PAFC mixture of pumping technique including 0.75% fibers had the greatest decrease in drying shrinkage, which was 29.5% lower than the plain mixture.

Mohammadhosseini et al. [15] examined the strength and transport properties of PAFC incorporated with polypropylene carpet fibers. In addition, cement was partially replaced by palm oil fuel ash. As a result of replacing 20% palm oil fuel ash with cement, the flowability of grout increased and the bleeding decreased. It was shown that introducing polypropylene carpet fibers to PAFC specimens reduced their compressive strength. PAFC specimen’s splitting tensile strength was significantly improved when fiber dosage of up to 0.75% was used. Nehdi et al. [16] investigated the mechanical properties of PAFC incorporated with short and long steel fibers. Results indicated that the specimens comprising 1% and 2% short steel fibers exhibited a compressive strength of around 14% and 18%, respectively, to the control specimen. Additionally, even at a high fiber dosage, the length of the steel fibers had only a negligible effect on the compressive strength of specimens. A multilayer PAFC slab with various steel fiber types and amalgamations was investigated by Murali and Ramprasad [17]. Three layers of 4, 2, and 4% multilayer PAFC were created and reinforced with three different fibers: crimped fiber, hooked end, and hybrid combination. Results indicated that the multilayered PAFC exhibited a superior impact resistance. Salaimanimagudam et al. [18] explored the impact performance of topology-optimized hammerhead pier concrete beam constructed using the idea of preplaced aggregate concrete. Steel fibers added to preplaced aggregate concrete increased the concrete’s ability to withstand impact loads by 22 to 40 times, according to Alfayez et al. [19]. Preplaced aggregate concrete that included tire rubber had a worse impact on performance.

In one set of beams, the whole cross-section was reinforced with steel fiber at 2 and 4%, while the other set of beams was only strengthened in the tension region with equal fibers. According to the findings, the completely fibrous concrete beam with 4% fiber was the most resistant to impact, with a first fracture and failure occurring at 2725 J and 3009 J, respectively, compared to the beam reinforced by fibers only in the tension zone. Ramkumar et al. [20] researched the layered PAFC comprising of a low carbon cementitious, steel fibers, clinker, calcined clay and fly ash. Results indicated that the number of hits causing initial cracking and failure was improved by about 507 and 1511%, respectively, compared to a non-fibrous specimen. Murali et al. [21] stated that the cracking impact number of multi-layered PAFC cylindrical specimens was raised by roughly 530–870%, with enhancement percentages exceeding 1350% during the failure stage of the specimens comprising long hooked end steel fibers, as compared to non-fibrous specimens. Projectile impact experiments on functionally graded PAFC mixes were carried out by Nandhu prasad et al. [22] to assess the projectile impact resistance of PAFC. According to the results, the compound bevel projectile needle has more impact resistance than the hollow edge and convex edge projectiles, irrespective of the fiber and distribution scheme used.

Much research was carried out to evaluate the impact resistance behavior of PAFC by using different materials, different configurations of fibers, glass fiber mesh and nanocarbon tubes. However, the research related to the impact resistance of different sizes of concrete beams was limited and needed special attention. In this research, the impact resistance of the PAFC beam with three different sizes was evaluated. Two different fiber types were used to reinforce the PAFC. In addition, analytical modeling was used to predict the failure impact energies and compared with the experimental findings. 

Several investigations have shown fiber schemes to influence concrete’s impact properties independently. As a result, there is no information in the technical literature on the development of concrete beams using the PAC concept. This research aims to determine the impact resistance of PAFC beams when subjected to drop weight. The influence of adding a new hybrid shape of crimp hooked end fibers on the impact behavior of PAFC beams of three sizes has yet to be determined. The three beam sizes were used to explore PAFC study parameters such as compressive strength, impact number (first crack and failure), ductility index, mode of failure and failure mechanism. The uniqueness of this study is in the development of a PAFC beam with a 3% fiber dose using the PAC principle and achieving improved impact resistance. In addition, an analytical model was used to compute the failure impact energies.

## 2. Experimental Study

### 2.1. Materials

**Figure 1:**
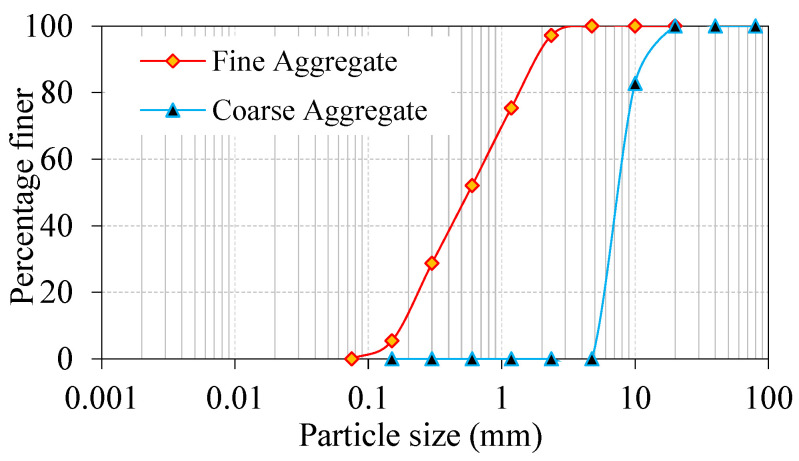
Aggregates particle size distribution.

**Figure 2:**
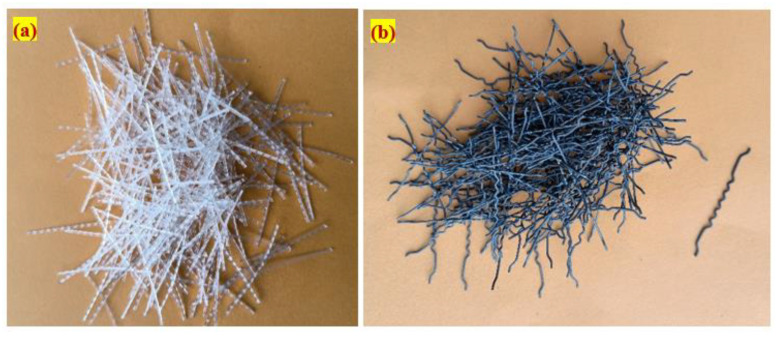
Used fibers in this research. (**a**) Polypropylene; (**b**) Steel.

This investigation used Ordinary Portland Cement as specified by IS: 12269-1987 [23], with a specific gravity of 3.14; the initial setting time observed was 33 min and final setting was 562 min, respectively. Additionally, the cement’s blain fineness was 369 m^2^/kg and the standard consistency was 31.2%. According to IS: 383-2016 [24], The fine aggregate was river sand, with a fineness modulus of 2.41, a specific gravity of 2.65, and a gradation curve that matched Zone II. To meet ASTM C939/C939M-16a [25] requirements, a grout with a particle size of less than 2.36 mm was used. As a result, the grout enters the aggregate and fiber skeleton with good gravity flow. As coarse material, we used natural gravel with a particle size of 12.5 mm. The coarse aggregates’ apparent bulk density was 1700 kg/m^3^, the water absorption was 0.56% and its specific gravity was 2.6. Figure 1 depicts the granulometric curve for aggregates utilized in this research. A commercialized admixture called Tech mix 640 was utilized in a plastic stage to increase the grouting time by reducing the quantity of mixing water needed. An additive that reduces water absorption is often included in a grout fluidifier, with the required dose of 1% by cement weight [26]. In this study, admixture doses were limited to 0.6% (cement weight) to meet flowability criteria, meet efflux durations, and minimize honeycombing. New fibers were employed to increase strength, including steel fibers (SF) with 50 mm lengths, 1 mm diameter, and 1150 Mpa tensile strength. Second, polypropylene fiber (PF) with a tensile strength of 500 Mpa with dimensions of 45 mm in length and 0.8 mm in diameter. Figure 2 shows the appearance of fibers that were employed in this experiment. The dosage of steel fiber in preplaced aggregate concrete is used up to 6% (by volume). In this study, fiber dosage is restricted to 3% due to the correlation of results with earlier publications that used 2–3% fibers. Since polypropylene fiber is a macro-type with low density, no issues were noted when 3% dosage was used during casting. More dosage of fibers can be used in preplaced aggregate concrete and this is challenging in traditional fiber-reinforced concrete.

### 2.2. Mixing Combination

In this experiment, the three mixtures were cast using a ratio (Binder to sand = 1.0 and water to binder = 0.42). For filling gaps in the aggregate structure, a water-reducing admixture and fine sand were utilized to make free-flowing cement grout. Several trials were employed to optimize these ratios and generate PAFC mixtures that satisfy the specified efflux time (35–40 ± 2 s). Based on their characteristics, two fibers were chosen. High tensile strength and density were essential factors in selecting the first fiber (SF). The second fiber’s low tensile strength and low density (PF) led to its selection. Three different mixtures and three different beam sizes were prepared with the same binder to sand and water to binder ratio. The first mixture was prepared without fiber and is considered a reference mixture designated as P-PC. The second mixture with a 3% dosage of steel fiber is designated as P-SFC. The third mixture with a 3% dosage of polypropylene fiber is designated as P-PFC. The three mixing combinations used in this research are demonstrated in Table 1.

### 2.3. Beam Preparation

**Figure 3:**
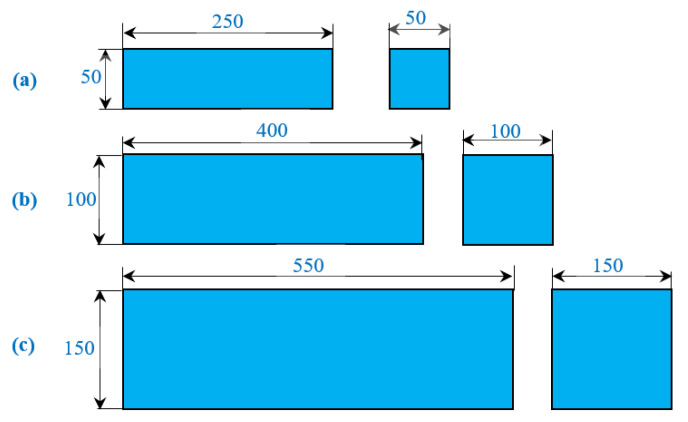
Three different beam geometries used in this research. (**a**) Small beam; (**b**) Medium beam; (**c**) Large beam.

**Figure 4:**
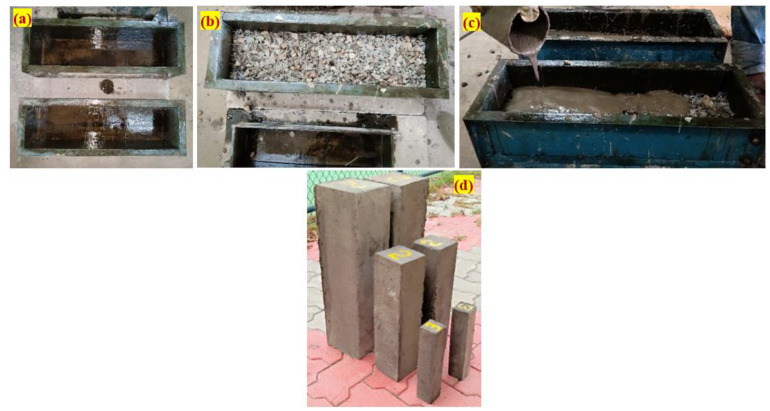
Casting procedure: (**a**) empty mold with oil coating; (**b**) packed fibers and aggregates; (**c**) grout injection; (**d**) demolded specimen.

As seen in Figure 3, three different beams were constructed to determine the impact strength of PAFC. The beams were designated as small beam (SB), medium beam (MB) and large beam (LB). The dimensions of the beams were selected from the literature [27,28]. Additionally, 100 mm PAFC cubes were made to determine the compressive strength. The following steps were included in the PAFC casting process: before adding coarse aggregate and fibers, oil was sprayed on all surfaces of the empty beam mold, as shown in Figure 4a. Following this process, natural skeleton layers were then created by packing in coarse aggregate and fibers into mold, as seen in Figure 4b. Cement grout was poured over the aggregate skeleton, as shown in Figure 4c. Moderate compaction was used to fill the concrete’s void spaces, and Figure 4d depicts the finished specimen. After casting, the specimens were permitted to rest in the molds for 24 h before the demolding process commenced, including immersion water curing for 28 days. The use of the PAC idea in the design of PAFC allows for the interlinking and packing of more coarse aggregates and fibers into the mold. Due to careful supervision, a strong, stable aggregate skeleton was formed. PAFC’s fresh state casting eliminated changing thickness and the impression of an undulating layer [29].

### 2.4. Compressive Strength and Drop Weight Impact Test

**Figure 5:**
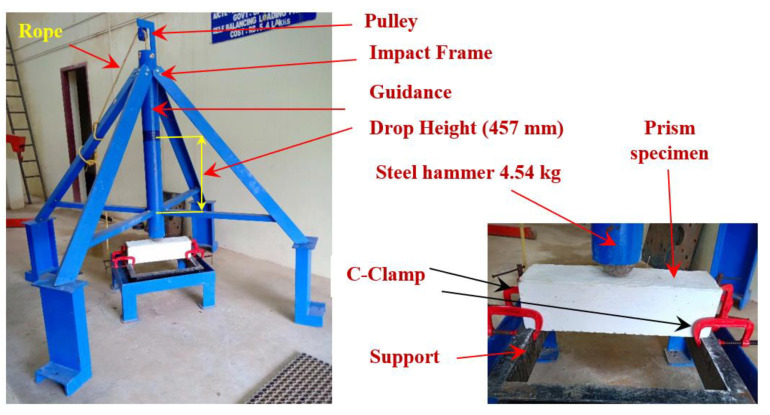
Impact testing device.

The compressive strength was evaluated using the 100 mm cubical specimens in conformity with IS: 516-2021 [30]. The average compressive strength of three specimens for the three distinct PAFC mixtures is used for the discussions. The impact energy absorption of PAFC beams was assessed using a repetitive drop weight impact test in conformity with ACI Committee 544 [31]. It is critical to note that the drop weight impact test is very simple since vibration, deformation data, or a time history are not required. Only the cracking and failure impact numbers need to be noted in this impact test. The ACI 544-2R repeated impact test equipment is made of a 4.54 kg free-falling steel mass that is released from a 457 mm height onto a specimen 152 mm in diameter and 63.5 mm in depth. Lifting the impact mass by hand and allowing it to fall naturally from the necessary height on a steel ball with a diameter of 63.5 mm is required. Stiff steel baseplates hold the specimen’s steel ball in place on the specimen’s top surface with the help of a unique steel frame. The steel ball acts as a shock absorber, transferring energy from the falling mass to the specimen’s top surface (See Figure 5). The specimen is repeatedly struck by manual operation until a noticeable surface break appears. Impact blows are counted as the cracking impact number (J1). The test is then repeated until the crack widens or the specimen is shattered such that it comes into contact with at least three of the four perimeter steel lugs, whichever occurs first. The procedure ends with the test being terminated, and the number of impacts is noted as the failure impact number (J2). Three cylindrical specimens were tested for each mixture and average impact energy was used for the discussion.

The measured impact energy by the beam defines the impact resistance, which may be computed using Equation (1).

(1) Impact energy=n×m×g×h

where *m* is the drop mass, *g* is the gravitational acceleration, *h* is the fall height and *n* is the impact numbers.

## 3. Results and Discussions

### 3.1. Compressive Strength of PAFC

**Figure 6:**
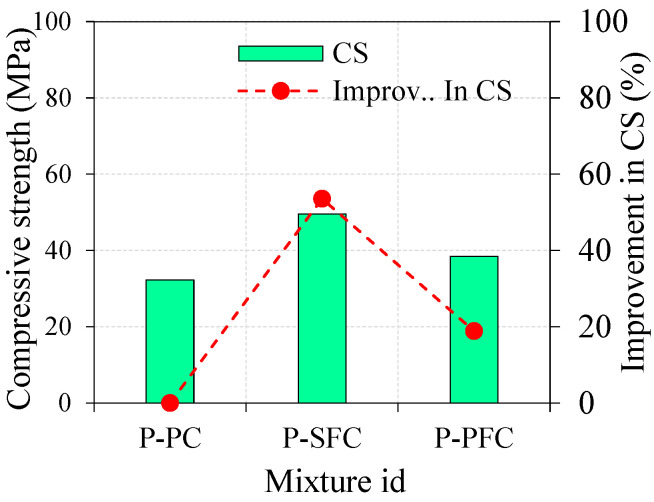
Compressive strength of PAFC.

The average compressive strength of the three specimens for the three distinct PAFC mixtures after 28 days is shown in Figure 6. The standard deviation of P-PC, P-SFC and P-PFC mixtures were 1.16, 2.45 and 3.25, respectively. In accordance with the findings shown in Figure 6, the compressive strength of the reference specimen was 32.3 Mpa. Adding SF to PAFC significantly enhanced compressive strength by 53.6% as associated with the reference specimen (P-PC). This phenomenon was due to SF’s existence, which had a remarkable capacity to bridge macro-cracks. Pulling out and debonding fibers became more difficult due to the crack’s convoluted route [12,32]. Adding PF influences the positive enhancement in compressive strength by about 18.9% compared to P-PC. One explanation for this behavior is that the cross-section had a consistent PF dispersion and was virtually 3D oriented, preventing large-scale cracks. This guaranteed that the stress was evenly distributed and that the crack course was altered, which led to fiber-bridging action that prevented the crack from progressing further [33]. The SF performs better than the PF because of its higher tensile strength, fiber-bridging action, and capacity to stop cracks from spreading [34]. In addition, the fibers perpendicular to the loading direction can increase the compressive strength because the fibers tend to confine the lateral expansion of the specimen, which reduces the crack propagation. Workability and consistent fiber dispersion are the primary reasons for limiting SF’s inclusion to typical fibrous concrete to 2% [16]. Since the compressive strength decreases with increasing fiber dosage, an increase of more than 2% causes fiber agglomeration and clustering, which in turn causes an increase in voids and weak spots. Prepacking fibers and coarse aggregate prior to grout application alleviates this issue with the benefit of the PAFC method [35].

Interestingly, PAFC mixtures were shown to have greater compressive strength. Increased fiber dose (3%) may be responsible for better resistance to crack creation and propagation, which leads to increased compression strength [36]. According to Nehdi et al. [16], the mixes containing 1% and 2% short steel fibers had compressive strengths that were 14% and 18% greater than those of the control specimens that did not include steel fibers.

### 3.2. Flexural Repeated Impact Test

#### 3.2.1. Effect of Fibers on the Impact Results of the Beam Specimens

**Figure 7:**
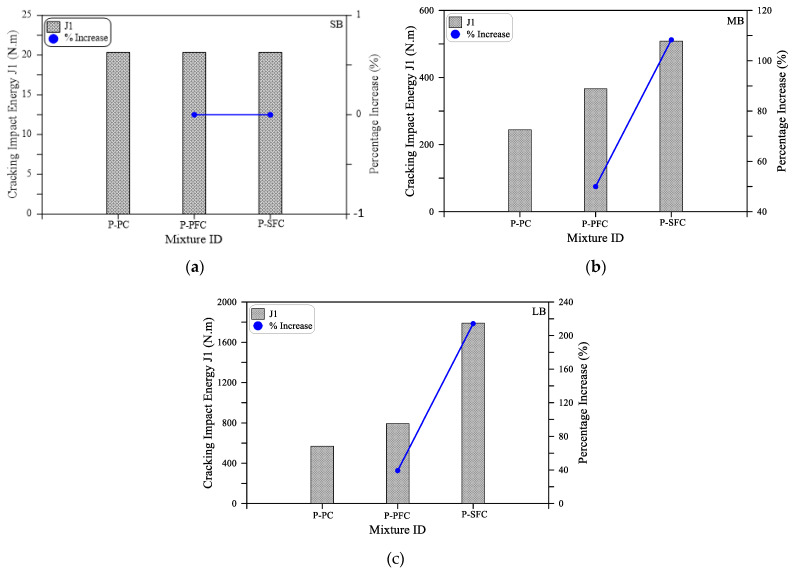
Cracking impact energy (J1) of the three mixtures: (**a**) SB; (**b**) MB; (**c**) LB.

**Figure 8:**
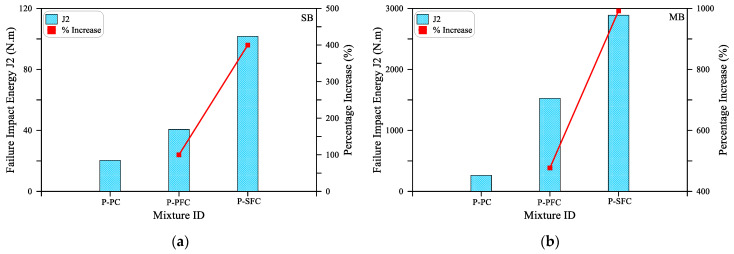

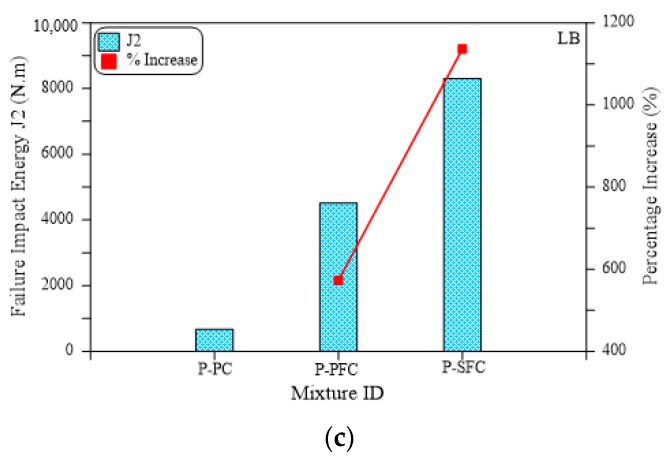
Failure impact energy (J2) of the three mixtures: (**a**) SB; (**b**) MB; (**c**) LB.

**Figure 9:**
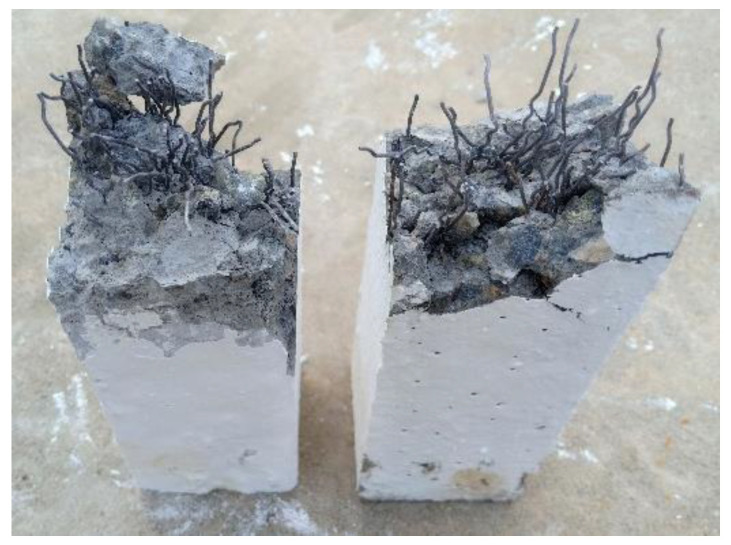
Failed steel fiber-reinforced specimens.

**Figure 10:**
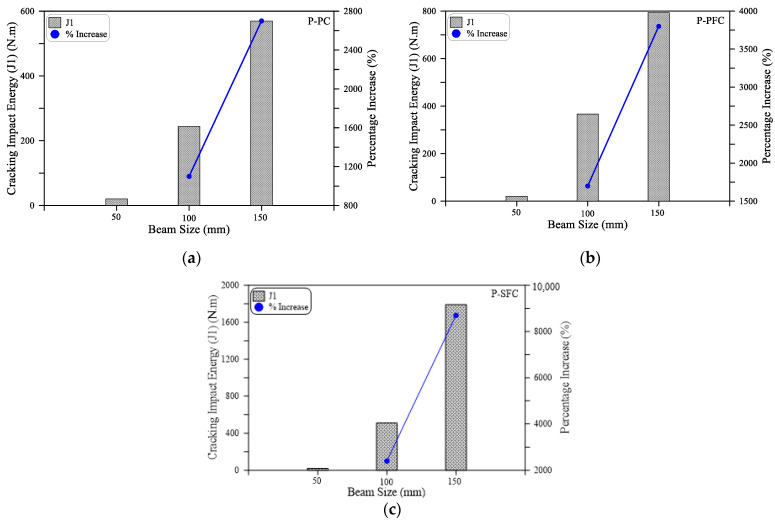
Cracking impact energy (J1) of the three beam sizes: (**a**) PC; (**b**) P-PFC; (**c**) P-SFC.

**Figure 11:**
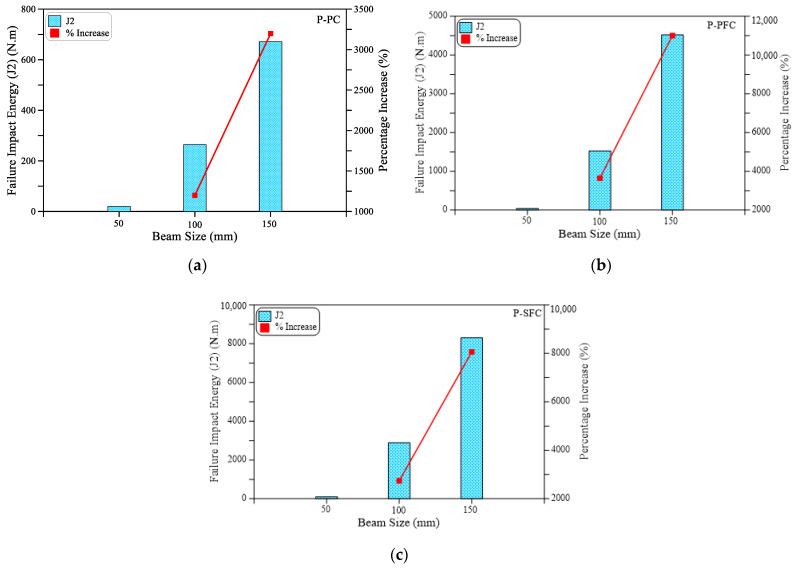
Failure impact energy (J2) of the three beam sizes: (**a**) PC; (**b**) P-PFC; (**c**) P-SFC.

The repeated drop weight flexural impact test findings are presented and discussed in this section. As disclosed in the previous section, two fibrous preplaced aggregate mixtures with steel and polypropylene fibers were prepared in addition to a third plain mixture. The impact results of the three mixtures for the three beam sizes are listed in Table 2 and presented in Figure 7, Figure 8, Figure 9, Figure 10 and Figure 11. The average results of the three specimens were presented in Table 2. It is clear from Table 2 that the observed minimum and maximum standard deviation of tested beams (SB, MB and LB) were 1.0 and 8.89, respectively. The standard deviation in statistics measures how widely distributed the results are. For example, a high standard deviation suggests an extensive range of values, whereas a low standard deviation shows a narrower range of possible values [37]. Rahmani et al. [38] reported that the standard deviation for the fibrous specimens tested under drop weight impact ranged from 35–69 for the first crack and from 36–90 for the failure, which indicates a higher standard deviation. However, the standard deviation values are near zero (<10% for the impact test), indicating less dispersion in test results [39]. In this study, the calculated standard deviation for all tested beams was less than 10%, indicating less dispersion in the test results. The impact results in Table 3 are presented in terms of the cracking impact energy (J1) and the failure impact energy (J2). The impact energy of each impact blow is simply calculated by multiplying the falling mass (w = 4.54 kg) times the ground acceleration (g = 9.81 m/s^2^) times the falling distance (h = 0.457 m). Hence, the impact energy of each impact blow equals 20.35 (N.m) or Joules. Thus, the impact energy equals 20.35 Joules times the retained number of impacts.

As shown in Figure 7a for the small size beam (SM), all mixtures retained the same impact cracking energy regardless of the type of mixture. Where the three beams with PF, SF and without fiber cracked after only one blow. On the other hand, the effect of the incorporation of fibers on the cracking impact energy is evident in Figure 7b,c for the medium size (MB) and large size (LB) beams. It is evident in the figures that the retained impact numbers of these beams till cracking are significantly higher than those of the small beam. It is also clear that the incorporation of PF increased J1 for the MB and LB beams by 50 and 39%, while by using SF, J1 increased by 108 and 214%, respectively. It should be noted that the percentage increase values of the specimens with PF fibers (P-PFC mixture) and SF fibers (P-SFC mixture) were calculated based on the corresponding retained values of the reference plain specimens (P-PC mixture). Similarly, the positive effect of incorporating polypropylene and steel fibers on the impact performance of preplaced aggregate concrete is clear in Figure 8, which depicts the retained impact energies of the three mixtures at failure. It is obvious in Figure 8a that the failure impact energy (J2) of the small beams increased by 100 and 400% when PF and SF, respectively, were used. On the other hand, Figure 8b,c show that the failure impact energy of the specimens with PF fibers was higher than the reference plain beams by 573 to 677% for the larger size beams, while this increased percentage jumped to 992 to 1136% for the specimens with steel fibers.

The positive effect of fibers on the impact resistance is due to their action as tiny reinforcing elements that help delay the widening of tensile cracks. Fibers bridge the two sides of the initiated cracks arresting its propagation and opening by withstanding the tensile stresses induced across the cracks under the repeated impact loads [40,41]. Therefore, the failure is postponed compared to plain specimens. It is also understood that the tensile strength of steel fibers is much higher than that of polypropylene fiber. Additionally, the rough surface and crimped configuration of SF assured a much better bond with the surrounding concrete than PF’s smooth surface. As the tensile stresses increased on the two sides of cracks, PF lost its bond faster than SF, while the hooked ends of the SF provided an additional bond strength [42] by the end anchorage inside the concrete, as shown in Figure 9. For the above-listed reasons, the retained impact energies of the P-SFC mixture with SF fibers were significantly higher than those of the P-PFC mixture with PF fibers. For instance, excluding the small size beams, the retained J1 records of the specimens with SF were approximately 1.4 to 2.3 times those of the specimens with PF. Similarly, J2 records of the specimens with SF were approximately 1.8 to 2.5 times those of the specimens with PF fibers. Another observable factor is that the fibers’ effect was generally more pronounced at the failure stage than at the cracking stage. Excluding the small size beam, the percentage increase in J1 due to fiber incorporation was in general between 39 and 214%, while the percentage increase in J2 was between 477 and 1136%. Comparing the percentage increase in J1 and J2 for each mixture and each beam size, the percentage increase in J2 for the larger size beams was in general 5.3 to 14.6 times the percentage increase in J1. The differences between the percentage increase at the two stages is attributed to the function of the fibers, where fibers become fully functional after the initiation of cracks, where the additional impact loads try to propagate and open these cracks, while the reinforcing fibers resist these trials by the bridging activity. On the other hand, fibers share a smaller amount of resisting tensile stresses when the cracks are still at the microscale and are not visible yet [43,44]. 

#### 3.2.2. Effect of Beam Size on the Impact Results of the Beam Specimens

The effect of specimen size on the flexural impact results of preplaced aggregate concrete is investigated in this section using the results of the small size beam (SB) with 50 mm cross-sectional side length, medium size beam (MB) with 100 mm cross-sectional side length and large size beam (LB) with 150 mm cross-sectional side length. The geometrical details of the three beams are depicted in Figure 4. The energy results in J1 and J2 of the three beam sizes are compared in Figure 10 and Figure 11 for the three mixtures P-PC, P-PFC and P-SFC. Figure 10 and Table 2 show that the cracking impact energy jumped by several times when the beam size increased from 50 mm to 100 mm. The retained J1 records of the 100 mm beams were 1100 to 2400% higher than their corresponding records of the 50 mm beams of the three mixtures. Similarly, for the three plain and fibrous mixtures, the recorded J2 values of the 100 mm beams were 1200 to 3650% higher than the corresponding records of the 50 mm beams. Increasing the beam size to 150 mm increased the retained number of impacts so that the percentage increase in J1 for the three mixtures dramatically jumped by 2700 to 8700% compared to the small beam SB. Similarly, J2 of the 150 mm beam exhibited percentage increases of 3200 to 11,000% over the corresponding 50 mm beams. The extraordinary increase in the retained impact numbers and hence the impact energy with the increase in beam size is an expected result. The reason is that the load is transformed into induced stresses in the material, while the load is kept constant (same drop weight and drop height); increasing the cross-sectional area would reduce the effective stress on the section. Hence, the stress induced by each impact blow becomes several times lower as the section size increases from 50 mm to 100 and 150 mm. Therefore, the material could resist significantly higher impact blows and absorb higher impact energy.

**Figure 12:**
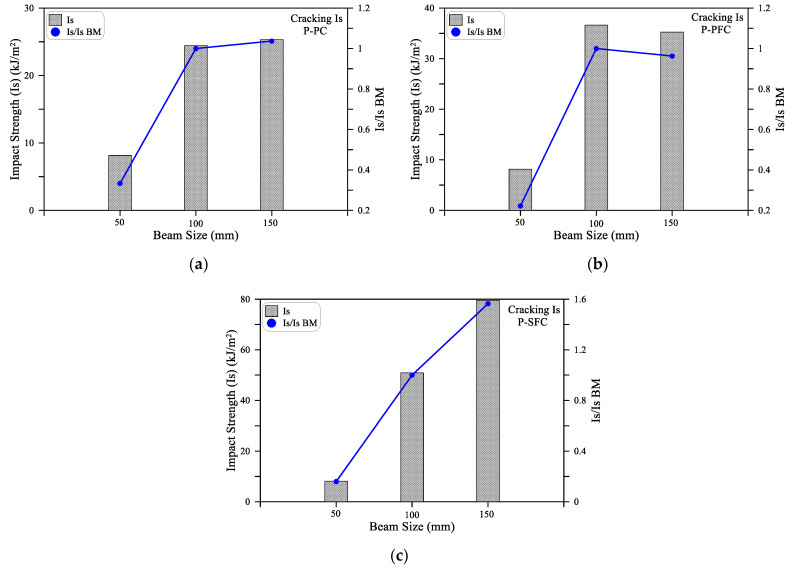
Cracking impact strength of the three beam sizes: (**a**) PC; (**b**) P-PFC; (**c**) P-SFC.

**Figure 13:**
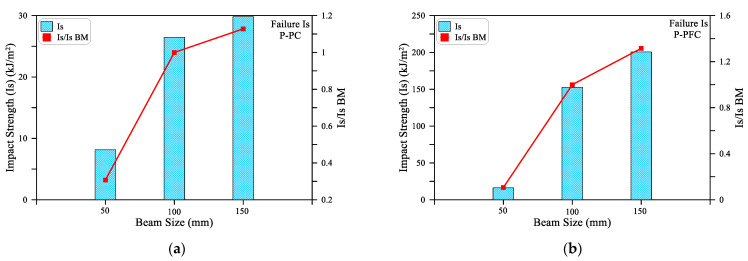

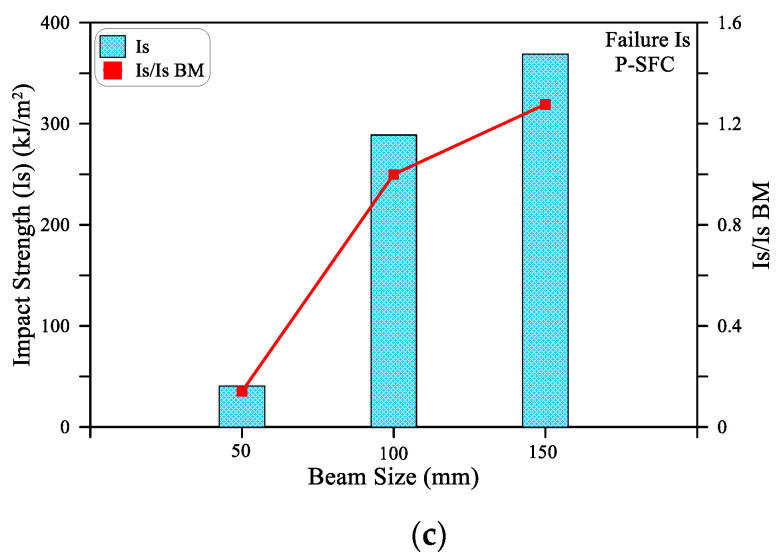
Failure impact strength of the three beam sizes: (**a**) PC; (**b**) P-PFC; (**c**) P-SFC.

To evaluate the effect of the cross-sectional area on the impact energy absorbed by the tested beam specimens, the Impact Strength (Is) is used here. The impact strength simply refers to the impact energy absorbed by a unit cross-sectional area of the tested beam. Hence, it can be said that it is the normalized impact energy by the cross-sectional area of the test specimen. This definition would provide a more fair tool to compare the impact resistance of beams with different cross-sectional areas. The impact strength (Is) is used in this section to compare the results of the small size beams with the cross-sectional side length of 50 mm, medium size beams (100 mm) and the large size beams (150 mm). Since the beam cross-section of 100 mm is the most widely adopted size for standard concrete material flexural tests, it was considered here as the reference to measure the decrease or increase in impact strength. Therefore, Figure 12 and Figure 13 show the Is values in addition to the ratios of the Is values of the small and large beams to the medium one (Is/Is BM). Table 4 shows the recorded Is values of the three beam sizes for the three mixtures. 

It is shown in Figure 12a,b that SB beams exhibited significantly lower impact strength values compared to MB and LB, which recorded approximately similar Is values. As shown in Table 4, for the P-PC mixture, the cracking impact strength was 8.1 kJ/m^2^ for the SB beam, while it was 24.4 and 25.3 kJ/m^2^ for MB and LB beams. The corresponding J1 values of the three beams were approximately 20, 244 and 570 J, respectively, revealing how this normalization (impact strength) is a useful comparison tool for impact strength of different size samples. The cracking impact strength of the LB beam of the P-PC and P-PFC mixtures was 0.96 to 1.03, while it was 1.56 for the P-SFC mixture. On the other hand, the SB was in the range of 0.16 to 0.33 for the three mixtures. Similarly, Figure 13 shows that the failure impact strength of SB was in the range of 0.1 to 0.3 of that of the medium size beam MB, while the large beam LB recorded failure impact strengths that were 1.13 to 1.31 times that of MB for the three mixtures. Thus, it can be said that the impact strength values of the medium- and large-size beams were comparable, while that of the small beam was much smaller. This result reveals that using beam specimens smaller than 100 mm would result in a noticeably underestimated evaluation of the impact strength under flexural impact. Increasing the size of the specimens larger than 100 mm leads to comparative evaluations with insignificant underestimation or small overestimation. Consequently, using beams with a cross-sectional side length of less than 100 mm is not recommended to evaluate the flexural impact strength. 

### 3.3. Impact Ductility of the Beam Specimens

Flexural impact ductility is a term that measures the capability of a beam to absorb impact energies after cracking till failure. This definition was interpolated from the flexural ductility, which measures the ability of a beam under flexure to absorb plastic energy before failure, which is calculated by the dividing of the deflation corresponding to the failure load (or a close load) to the deflection corresponding the yield load [45], where steel bar yielding refers to the end of the service stage and the initiation of the plastic stage. Since plain concrete specimens do not exhibit a yielding stage, the cracking load can be considered the point that changes the specimen behavior from elastic to plastic. Therefore, the impact ductility index can thus be stated as the ratio of impact energy at the failure stage (J2) to the impact energy at the cracking stage (J1).

**Figure 14:**
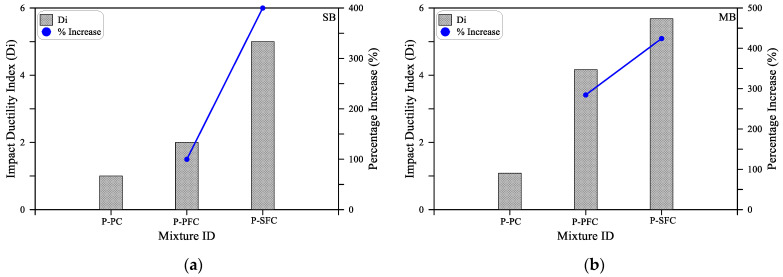

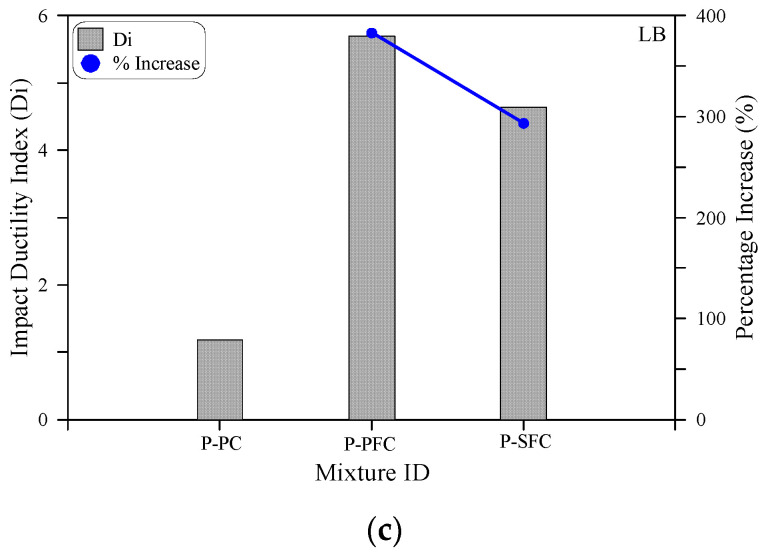
Impact ductility index (Di) of the three mixtures: (**a**) SB; (**b**) MB; (**c**) LB.

The impact ductility index is depicted for the three beam sizes in Figure 14. It is understandable in the figure that the fibrous beams showed a higher ductility index compared to the plain mixture, regardless of the beam size. Whereas for the SB beams, the ductility index of the beams with PF and SF were, respectively, 100 and 400% higher than that of the plain beams of the mixture P-PC. Similarly, the ductility index of the beams with PF and SF fibers was higher by 285 and 424% compared to the plain specimens for the MB beams. On the other hand, for the large beams, percentage increase values of 383 and 293% were recorded for beams with PF and SF fibers compared to the plain beams. The increase in the ductility is directly attributed to the crack propagation and widening alteration gained by the crack bridging activity of fibers, which delayed the failure to several more impact blows and extended the specimens’ plastic resistance. 

### 3.4. Mode of Failure

**Figure 15:**
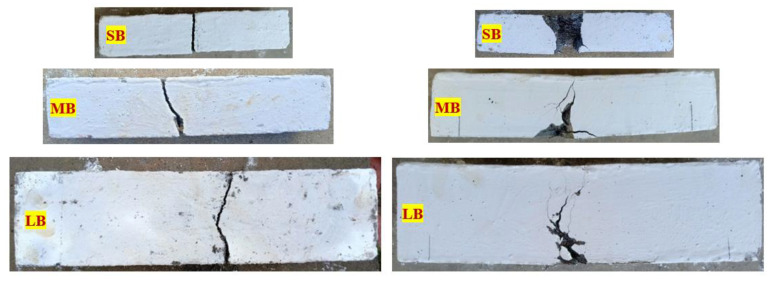
Failure mode of PAFC beams.

Figure 15 displays all reference beams’ severely damaged brittle failure (SB, MB, and LB). Because of the lack of bridging actions and concrete’s brittle nature, the beams broke into two parts owing to the lack of energy dissipation and its inability to restrict crack progression. In all three P-PC beams, the brittle behavior was evident after cracking had been induced. Initially, the specimen’s bottom surface had a hairline crack, but the crack became wide enough to separate it into two parts following repeated impacts. According to the literature, this form of brittle failure is predicted in a non-fibrous beam in good agreement [46]. Conversely, all PAFC beams displayed a greater capacity to absorb impact energy, higher ductile response, and the ability to restrict crack propagation due to the higher dosage of fiber. Figure 15 depicts the failure mode of the fibrous beams. A first microcrack appears at the bottom surface of the beam, which later becomes wider and extends to its top surface. In this stage, crack propagation was deferred to the top surface because the fiber and matrix were well-bonded and the fibers were better able to withstand deformation.

### 3.5. Modeling of Collision Energy of MLPAFC

The influence of three factors controls the failure impact energies: (i) matrix cracking, (ii) matrix/fiber debonding, and (iii) fiber sliding. The fibers and matrix unitedly withstand falling mass impact in the initial loading action phase. In the instant case, stress transmission occurs from the matrix to fiber through the boundary of matrix/fiber [47]. However, stress transmission happens in the fibers alone when cracks are initiated in the matrix due to augmented load at the corresponding point. The matrix/fiber delaminates and a slip-off happens when the inconsistency between the fibers and matrix reaches a dangerous value [47,48]. Deformation continues, resulting in fiber slip-off from the matrix, leading to damage. Despite fiber pull-off progression entailing three progressions: matirx/fiber work collaboration, delamination of fiber from matrix, and matrix/fiber slip, the last two are amalgamated as one. Matrix/fiber delamination is the initial phase in fiber slip-off. Shear bond strength is equal to matrix/fiber interfacial shear strength [49,50]. In accordance with the mixing rule of thumb, impact energy engrossed by the specimens is articulated in Equation (2) [47].

(2) $$E=E_{1}V_{m}+F_{1}E_{2}$$

where *E* and *E*_1_ imply total engrossed collision energy by the fibrous and non-fibrous specimens, respectively, *V_m_* denotes matrix volume fraction, *F*_1_ implies fibers in numbers that are visible in the crack plane and can be assessed in Equation (3) and *E*_2_ is the energy from each fiber.

(3) $$F_{1}=\frac{K_{a}V_{f}}{\pi r^{2}}=\frac{4K_{a}V_{f}}{\pi d^{2}}$$

where *K_a_* is the area crack plane of the MLPAFC specimens, *V_f_*_is_ the dosage of steel fibers in MLPAFC, and *d* and *r* are the used fiber diameter and radius. The efforts undertaken to obtain the solitary fiber out of the matrix are in accordance with the following procedure. Assuming that the fiber has a diameter at a distance of *x* in opposition to interfacial shear stress *τ_i_* the total force resistant to the fiber getting out of the matrix at the instant on the delaminated fiber surface is *τ_i_πd* (*k*–*x*), here *k* implies the embedded length of the fiber. When the fiber further obtains a length of dx, the work carried out by this force is τiπd (*k*–*x*) *dx*. The work in getting the fiber out through a distance *k* is attained by integrating it as Equation (4). Therefore,

(4) $$E_{2}=\overset{l/2}{\underset{0}{\int }}\tau_{i}\pi d\;\left(k-x\right)dx=\frac{\tau_{i}\pi dk^{2}}{2}$$

The fiber length from the matrix may deviate in a minimum and maximum range of 0 and *l*/2, respectively, where *l* implies critical fiber length. Hence, the mean of each fiber pulled out is attained via integration of *dk*, which yields Equation (5)

(5) $$E_{1}=W_{fp}=\frac{1}{l/2}\overset{l/2}{\underset{0}{\int }}\frac{\tau_{i}\pi dk^{2}}{2}dk=\frac{\tau_{i}\pi dl^{2}}{24}$$

where *W_fp_* is energy per fiber.

Therefore,

(6) $$E_{2}=W_{fp}=\frac{\tau_{i}\pi dl^{2}}{24}$$

Substituting Equation (6) into Equation (2) gives:

(7) $$E=E_{1}V_{m}+\frac{\tau_{i}l_{1}^{2}K_{a}V_{f}}{6d_{1}}$$

To find the engrossed impact energy from Equation (7), it is essential to attain *τ_i_*, which describes the matrix/fiber friction. The *τ_i_* value can be directly obtained from Equation (8) as long as flexural stress is attained through the flexural strength test [51,52].

(8) $$\sigma =\frac{1}{2}V_{f}\;g\;\tau_{i}\left(\frac{L_{f}}{d_{f}}\right)+\sigma_{m}\left(1-V_{f}\right)$$

where *σ* is the flexural stress of the fibrous specimen and, *σ_m_* is the flexural strength of the reference specimen, *g* = 1.5 [53].

The obtained theoretical results from Equation (7), in conjunction with the mean values attained from the experimental test, are given in Table 5, revealing that the modeling results compare well with the experimental results. For the SB, the percentage difference between the experimental and modeling results by 9 and 11.9% for the P-SFC and P-PFC beams, respectively. The modeling results of SB were overestimated for both fibrous specimens. In the case of MB and LB beams, these percentage differences ranged from -2.1 to 6.6%, which indicates the modeling results were underestimated irrespective of fiber type and specimen size. The minimum and the maximum percentage difference between the experimental and modeling values were −2.1 and 11.9%, respectively, which indicates the acceptable limit of less than 20% difference suggested by IS:456-2000 [54]. Yu et al. [55] reported that the modeling results were (about 9.3%) higher than the experimental results for the smaller specimens, attributed to the energy dissipated into the testing device.

The model significantly overestimates the experimental results for SB and this trend is reversed for MB and LB. Because the test device vibration or the friction between the specimen and the instrument is not taken into account in the modeling process, this might be the cause. In fact, when the concrete’s impact resistance capacity is quite large, tiny vibrations of the drop weight impact device may be observed, which indicates that some of the energy is dissipated in the equipment.

This model has a few limitations: (1) interfacial bond strength can be calculated from the flexural strength. Therefore, the single loading point flexural strength value should be used to find interfacial bonding since impact loading is also a single point. Two-point loading flexural strength does not apply to this model; (2) the lower fiber dosage (below 0.6% by volume), the testing data have a good agreement with the experimental results, according to Xu et al. [47]. A higher fiber dosage of 3% used in this study leads to an 11.9% difference between experimental and modeling results. A further study is required to set the limitation for the fiber dosage.

## 4. Conclusions

The following conclusions can be drawn from the impact test results obtained from the preplaced aggregate concrete beams tested in this study under repeated flexural impact loading.

1. The incorporation of fibers led to increased flexural impact energy absorption capacity. This phenomenon is due to the higher tensile strength of steel fibers (SF) with higher resistance to crack propagation than polypropylene fibers (PF). Considering the larger size beams, the cracking impact energy (J1) improved by 39 to 50% when PFs were incorporated, while the incorporation of SF led to an increase in J1 of 108 to 214% compared to the plain beams;
2. The effect of fibers on the impact energy of the tested beams was much more pronounced at the failure stage than at the cracking stage, which is attributed to the main function of fibers as discrete reinforcing elements across cracks. Considering the larger size beams for all mixtures, the comparison of the percentage increase in impact energy at the cracking stage and the failure stage (J2) revealed that J2 was higher by 5.3 to 14.6 times than J1;
3. The impact strength, representing the recorded impact energy normalized by the cross-sectional area of the tested beam, showed that the beams with side lengths of 100 and 150 mm exhibited approximate strengths, while the recorded impact strengths of the small beams (50 mm) were significantly lower. Thus, it was concluded that small-size beams (smaller than 100 mm) are not recommended for flexural impact tests as they may noticeably underestimate the material’s impact strength;
4. The presence of PF and SF fibers in the mixtures enabled the beams to withstand higher tensile stresses across the cracks owing to the bridging activity of the fibers, which extended the failure to several more impacts after cracking. Therefore, the retained J2 of the fibrous specimens were much higher than their corresponding J1 records. As a result, the impact ductility index of the fibrous specimens was several times higher than that of plain beams. For the larger size beams and for all mixtures, the impact ductility index of the fibrous beams was 285 to 424% higher than the corresponding ductility index of plain beams;
5. The failure mode of all non-fibrous beams, regardless of size, was brittle, while the ductility failure was recorded in all of the fibrous beams. The failure impact energy recorded from experimental and computed were close to each other, which is evidence that the modeling results are accurate;
6. This model has a few limitations: (1) interfacial bond strength can be calculated from the flexural strength. Therefore, the single loading point flexural strength value should be used to find interfacial bonding since impact loading is also a single point. Two-point loading flexural strength does not apply to this model; (2) the lower fiber dosage (below 0.6% by volume) and the testing data have a good agreement with the experimental results.

## Figures and Tables

**Table 1:** Mixing combinations.

Mixture ID	Mix Proportion of Grout for Producing 1 m^3^			Coarse Aggregate (kg/m^3^)	Fiber Type	Fiber Dosage (%)	Superplasticizer (%) by Cement Weight
	Cement (kg)	Sand (kg)	W/C				
P-PC	800	800	0.42	1448	-	-	0.4
P-SFC	800	800	0.42	1448	Steel	3	0.6
P-PFC	800	800	0.42	1448	Polypropylene	3	0.6

**Table 2:** Impact strength results of the tested beams.

Mixture ID	Impact Number						Standard Deviation					
	SB		MB		LB		SB		MB		LB	
	N1	N2	N1	N2	N1	N2	0	0	1.53	1.53	3.06	4.51
P-PC	1	1	12	13	28	33	0	1	3.51	7.77	8.33	8.08
P-PFC	1	2	18	75	39	222	0	0	2.65	8.89	4.58	8.02
P-SFC	1	5	25	142	88	408	0	0	1.53	1.53	3.06	4.51

**Table 3:** Calculated impact energies of the tested beams.

Impact Energies					
SB		MB		LB	
J1	J2	J1	J2	J1	J2
20.35	20.35	244	264.49	570	671.39
20.3	40.69	366	1525.88	793	4516.59
20.3	101.73	509	2888.99	1790	8300.76

**Table 4:** Impact strength of the tested beam specimens.

Beam Size	Impact Strength is (kJ/m^2^)					
	P-PC		P-PFC		P-SFC	
	Is1	Is2	Is1	Is2	Is1	Is2
SB	8.1	8.1	8.1	16.3	8.1	40.7
MB	24.4	26.4	36.6	152.6	50.9	288.9
LB	25.3	29.8	35.3	200.7	79.6	368.9

**Table 5:** Impact energy comparison (experimental and computed values).

Mixture ID	Impact Energy (J) for SB		Impact Energy (J) for MB		Impact Energy (J) for LB	
	Experimental	Computed	Experimental	Computed	Experimental	Computed
P-SFC	101.73	110.88	2888.99	2697.22	8300.76	8058.46
P-PFC	40.69	45.51	1525.88	1493.16	4516.59	4240.49

## Data Availability

Not applicable.
